# Hedgehog Pathway Inhibition by Novel Small Molecules Impairs Melanoma Cell Migration and Invasion under Hypoxia

**DOI:** 10.3390/ph17020227

**Published:** 2024-02-08

**Authors:** Alessandro Falsini, Gaia Giuntini, Mattia Mori, Francesca Ghirga, Deborah Quaglio, Antonino Cucinotta, Federica Coppola, Irene Filippi, Antonella Naldini, Bruno Botta, Fabio Carraro

**Affiliations:** 1 Cellular and Molecular Physiology Unit, Department of Molecular and Developmental Medicine, University of Siena, 53100 Siena, Italy; alessandro.falsini@student.unisi.it (A.F.); gaia.giuntini@student.unisi.it (G.G.); federica.coppola@student.unisi.it (F.C.); irene.filippi@unisi.it (I.F.); antonella.naldini@unisi.it (A.N.); 2 Department of Biotechnology, Chemistry and Pharmacy, University of Siena, 53100 Siena, Italy; mattia.mori@unisi.it; 3 Department of Chemistry and Technology of Drugs, Sapienza University of Rome, 00185 Rome, Italy; frncesca.ghirga@uniroma1.it (F.G.); deborah.quaglio@uniroma1.it (D.Q.); bruno.botta@uniroma1.it (B.B.); 4 Department of Molecular Medicine, Sapienza University, 00161 Rome, Italy; antonino.cucinotta@uniroma1.it; 5 Cellular and Molecular Physiology Unit, Department of Medical Biotechnologies, University of Siena, 53100 Siena, Italy

**Keywords:** hypoxia, Hedgehog pathway, carbonic anhydrases, chemical compounds

## Abstract

Melanoma is the principal cause of death in skin cancer due to its ability to invade and cause metastasis. Hypoxia, which characterises the tumour microenvironment (TME), plays an important role in melanoma development, as cancer cells can adapt and acquire a more aggressive phenotype. Carbonic anhydrases (CA) activity, involved in pH regulation, is related to melanoma cell migration and invasion. Furthermore, the Hedgehog (Hh) pathway, already known for its role in physiological processes, is a pivotal character in cancer cell growth and can represent a promising pharmacological target. In this study, we targeted Hh pathway components with cyclopamine, glabrescione B and C22 in order to observe their effect on carbonic anhydrase XII (CAXII) expression especially under hypoxia. We then performed a migration and invasion assay on two melanoma cell lines (SK-MEL-28 and A375) where Smoothened, the upstream protein involved in Hh regulation, and GLI1, the main transcription factor that determines Hh pathway activation, were chemically inhibited. Data suggest the existence of a relationship between CAXII, hypoxia and the Hedgehog pathway demonstrating that the chemical inhibition of the Hh pathway and CAXII reduction resulted in melanoma migration and invasion impairment especially under hypoxia. As in recent years drug resistance to small molecules has arisen, the development of new chemical compounds is crucial. The multitarget Hh inhibitor C22 proved to be effective without signs of cytotoxicity and, for this reason, it can represent a promising compound for future studies, with the aim to reach a better melanoma disease management.

## 1. Introduction

### 1.1. Melanoma, TME and Associated Signalling Pathways

Melanoma is known to be one of the deadliest kinds of tumour due to its ability to generate metastasis, and its incidence in the population is increasing [1]. Besides genetic predisposition and other environmental pathways, UV exposure is considered to be the main source of mutations that arise [2]. BRAF mutations are frequently associated with melanoma onset and BRAF inhibitors have been long studied, but pharmacological resistance to these treatments has been reported [3]. In this context, the production, development and testing of new small molecules can be crucial to overcome drug resistance by targeting other signalling pathways which are involved in tumour progression [4]. Indeed, the main problem related to this form of skin cancer is its ability to migrate and invade other body sites [5].

First discovered in Drosophila melanogaster, the canonical Hh pathway relies on Hh ligand interaction with Patched that allows Smoothened (SMO) activation and this, after intermediate steps, determines GLI1 activation and translocation into the cell nucleus with the result of target gene transcription [6]. In addition, GLI1 can be activated by other oncogenic pathways (such as PI3K/AKT, RAS and MEK/ERK) without SMO involvement, but with the result of GLI1 target gene activation [7]. Furthermore, the non-canonical Hh pathway (type I) which is SMO-independent is active without the activation of GLI1 (Hh binding disrupts the interaction of PTCH with cyclin B1, leading to increased proliferation and survival); on the other hand, we find the non-canonical Hh pathway (type II) which depends only on SMO (SMO-dependent) characterised by enhancement of Ca^2+^ oscillations and cytoskeleton regulation [8,9]. The Hh pathway plays different roles during the lifespan of an organism, as it is involved in embryonic development and adult organism homeostasis (adult stem cells), but it is aberrantly activated in cancer cells, promoting tumour progression [10].

Furthermore, the tumour microenvironment (TME) contributes to melanoma progression since it is characterised by hypoxia (and therefore by a low O_2_ tension). Indeed, the hypoxic condition is known to enhance melanoma migration and invasion. As a matter of fact, metastatic melanoma, which migrates and invades, has to adapt to the initial hypoxic phase [11,12] by the accumulation of hypoxia inducible factor 1α (HIF-1α) and the transcription of its target genes [13].

Moreover, carbonic anhydrases (CAs) are known to be relevant in cancer cell migration and invasion ability [14] and they are associated with the hypoxic TME. CAs are a family of enzymes expressed in all living organisms from bacteria to plants and animals. Of all the eight classes known nowadays, only α-CAs are present in animals, and only sixteen play a pivotal role in humans, which are involved in physiological and pathological processes [15]. CAs catalyse the reversible hydration of carbon dioxide (CO_2_) to bicarbonate (HCO_3_^−^) and protons, acting as a pH regulator. Of interest, CAIX and CAXII are related to hypoxia and to melanoma cell migration and invasion, but less is known about CAXII compared to CAIX.

### 1.2. Why Chemical Compounds?

We have previously demonstrated that CAXII is downregulated by SMO and GLI1 siRNA, resulting in melanoma migration and invasion impairment [16]. Moreover, the correlation between melanoma development, Hh signalling, and CAs is increasingly highlighted by recent studies [17] and targeting this pathway could represent a strategy to reduce melanoma severity. Therefore, we focused our attention on testing new chemical compounds in vitro in order to confirm the link between the Hh pathway, hypoxia, and CAXII [10] and to increase our knowledge on small molecule activity, which can be further developed as promising drugs in melanoma treatment.

In this study, we used different chemical compounds: cyclopamine, a plant-derived steroid alkaloid and well-known SMO inhibitor; Smoothened agonist (SAG), which directly binds to SMO and results in the activation of the Hh pathway; glabrescione B (GlaB), an isoflavone naturally found in the seeds of Derris glabrescens (Leguminosae) and a direct GLI1 inhibitor [18]. In particular, GlaB was identified as a potent inhibitor of GLI1/DNA interaction, with strong anticancer efficacy against Hh-dependent cancers such as basal cell carcinoma and medulloblastoma (MB). The high versatility of the isoflavone scaffold has been further exploited for targeting the Hh signalling pathway at multiple levels, and the GlaB derivative C22 was identified as the first and most efficient multitarget Hh inhibitor that simultaneously targets both SMO and GLI1 [19]. C22 showed strong inhibitory properties on Hh signalling when tested in functional and biological in vitro assays and in an in vivo model of Hh-dependent MB, without signs of cytotoxicity [19]. Compared to siRNA, small molecules are endowed with a higher potential for drug development mostly due to their enhanced chemical stability in different organism compartments, increased ability to be delivered to the desired site and ability to cross biological membranes [20].

Therefore, we decided to test these Hh inhibitors in vitro in order to target SMO and/or GLI1 and analyse melanoma migration and invasion to see if they could reduce this activity as a first step in developing new therapeutical strategies for melanoma treatment.

## 2. Results

### 2.1. HIF-1α and SMO Kinetics; CAXII Modulation by Known Hh-Interfering Molecules

Since hypoxia is able to modulate melanoma cell migration and invasion, we focused on the pathways that may be involved. Firstly, we analysed by Western blotting HIF-1α accumulation in both cell lines exposed either to normoxia or hypoxia.

**Figure 1:**
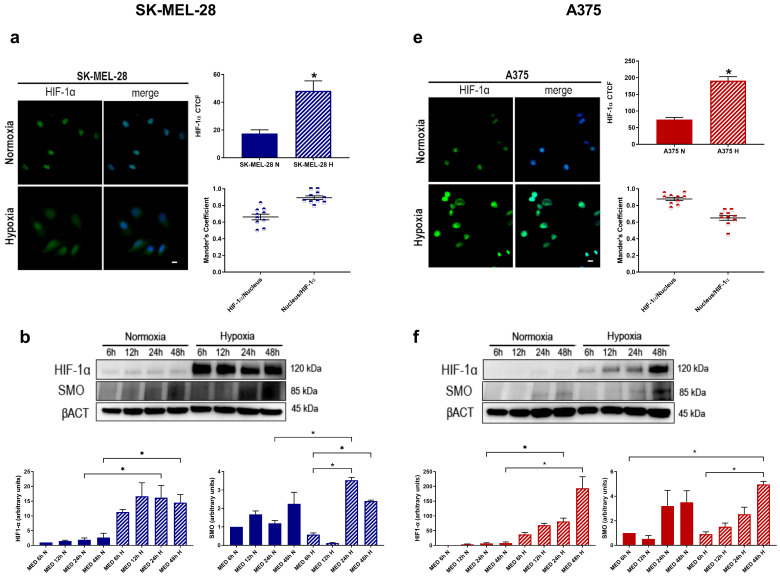

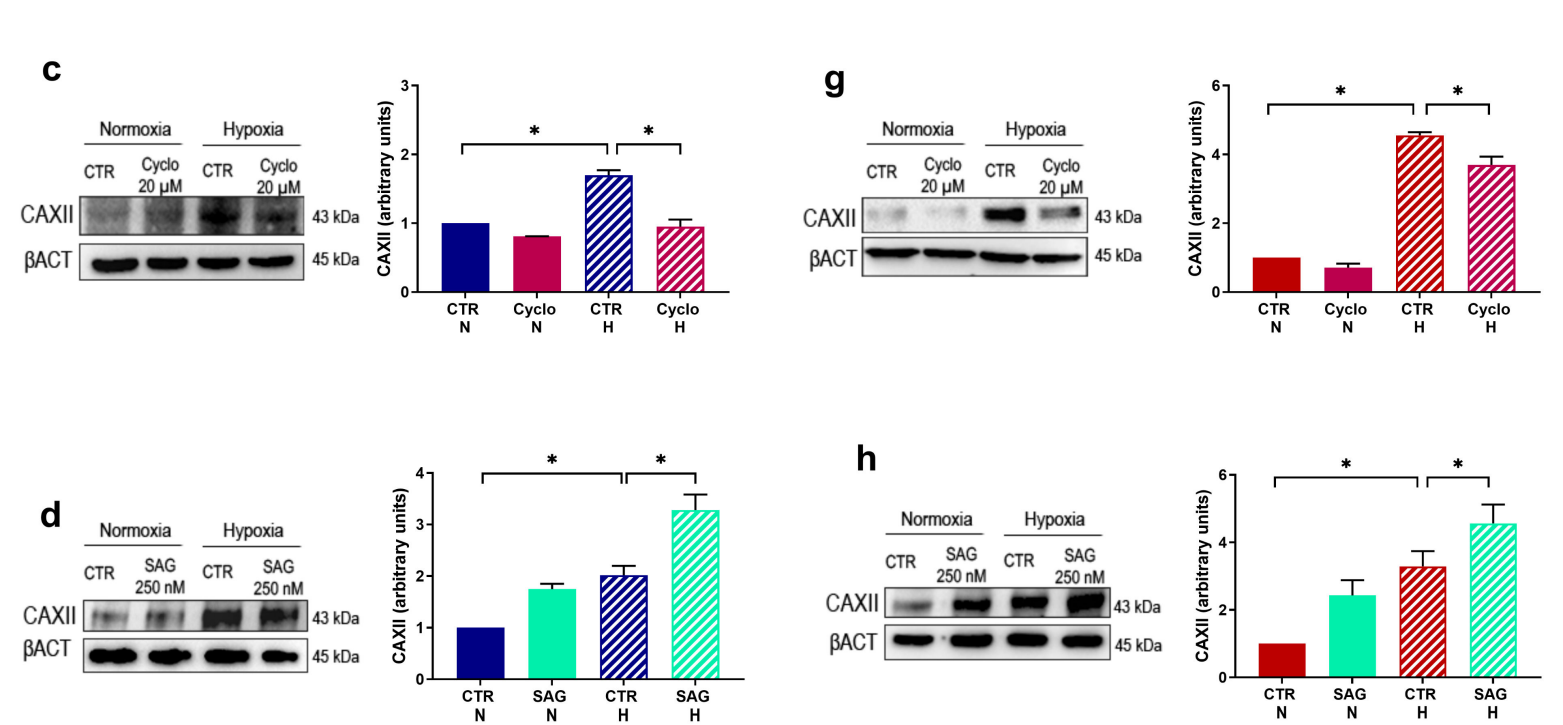
HIF-1α and SMO kinetics; CAXII expression after treatment with known Hh-interfering molecules in melanoma cell lines. HIF-1α and SMO (**b**–**f**) Western blot kinetics (6 h, 12 h, 24 h and 48 h) under normoxic or hypoxic conditions. β-actin was used as loading control. Blots are representative of three independent experiments. HIF-1α levels detected by immunofluorescence (**a**–**e**) (20× magnification, scale bar 10 μm) in SK-MEL-28 and A375 under normoxia and hypoxia. Colocalisation analysis of HIF-1α and cell nucleus expressed by Mander’s coefficient as mean ± SEM (10 cells/sample) indicating 0 (absence of colocalisation) and 1 (maximum of colocalisation); means ± SEM are presented. (*n* = 3; * *p* ≤ 0.05 indicates statistically significant difference). CAXII Western blot after treatment with cyclopamine (**c**–**g**) or SAG (**d**–**h**) under normoxic or hypoxic conditions for 24 h. β-actin was used as loading control. Blots are representative of three independent experiments. Means ± SEM are presented. (*n* = 3; * *p* ≤ 0.05 indicates statistically significant difference). CTR = control, N = normoxia, H = hypoxia.

As shown in Figure 1b–f, a significant increase in HIF-1α accumulation was reported under hypoxic conditions at all time points (6 h, 12 h, 24 h and 48 h), compared to the relative normoxic controls, in both cell lines. In order to enhance these data, we performed immunofluorescence analysis at 24 h in SK-MEL-28 and A375 (a–e) which revealed a significantly increased HIF-1α signal under hypoxia. Furthermore, in this condition, we showed a colocalisation between HIF-1α and the cell nucleus, which was labelled with Hoechst. Therefore, we concluded that HIF-1 was transcriptionally active in our melanoma cell lines under hypoxia. Concerning the same kinetics end-time, we analysed SMO protein levels by Western blot analysis (b–f): we noticed an increased trend of SMO protein under hypoxic conditions, at 24 h and 48 h of hypoxia treatment, in both cell lines.

Furthermore, we evaluated CAXII expression after treatment with cyclopamine and SAG, in both cell lines under normoxia and hypoxia, in order to understand if there is a possible link between CAXII and the Hh pathway. As represented in Figure 1c–g, by Western blot images, we observed a significantly increased CAXII expression in hypoxia with respect to the normoxic control in both cell lines. Treatment with cyclopamine significantly reduced CAXII protein level under hypoxia in SK-MEL-28 (c) and in A375 (g), while SAG enhanced CAXII expression in normoxia and more evidently under hypoxia in both cell lines (d–h). Notably, despite the increased CAXII determined by hypoxia, treatment with SAG resulted in an even higher expression of CAXII. CAXII is modulated by Hh-interfering molecules, revealing a possible interplay between the Hh pathway and CAXII expression.

### 2.2. GLI1 and SMO Protein Level Is Impaired by GlaB and C22 Treatment

We then took into account two new Hh pathway inhibitors, GlaB and C22, and we tested their ability to inhibit the Hh signalling pathway in melanoma cells under hypoxic conditions. We first perform a cytotoxic assay without any significant sign of cytotoxicity in our cellular model, according to the work by Lospinoso et al. [19]. Then, we treated SK-MEL-28 and A375 cell lines with 1 μM of our compounds. The impact of the pharmacological treatment was monitored by Western blot analysis and confirmed by immunofluorescence.

**Figure 2:**
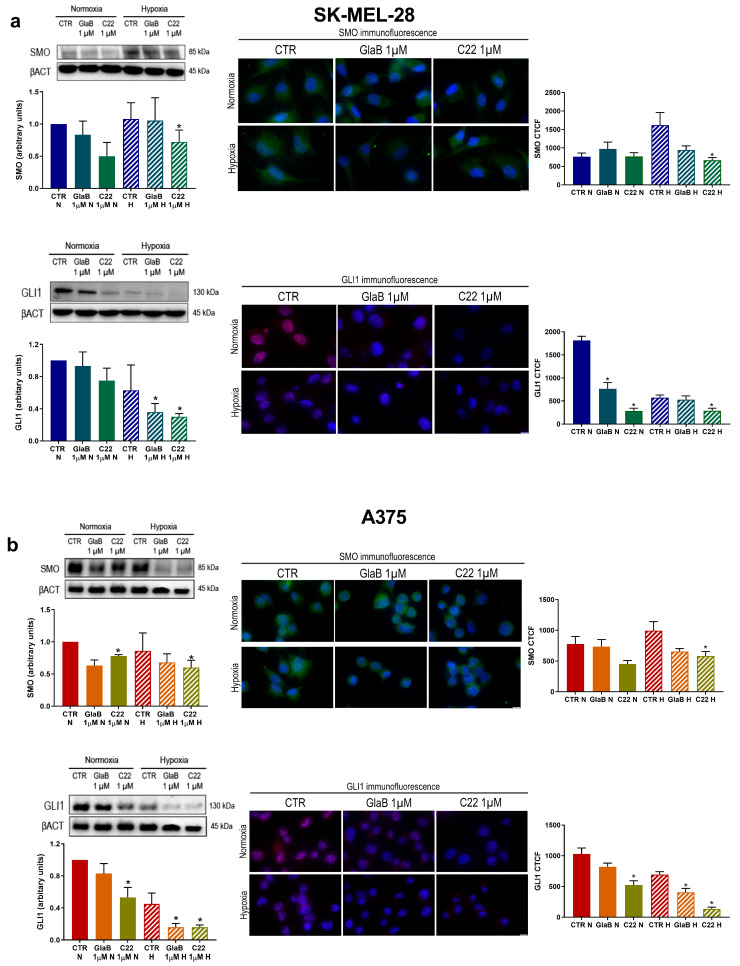
SMO and GLI1 expression after chemical treatment. GLI1 and SMO Western blot and immunofluorescence (60× magnification, scale bar 10 μm) after treatment with GlaB or C22 in SK-MEL-28 (**a**) and A375 (**b**) under normoxia and hypoxia for 24 h. β-actin was used as loading control for Western blot. Blots are representative of three independent experiments. Means ± SEM are presented. (*n* = 3; * *p* ≤ 0.05 indicates statistically significant difference). CTR = control, N = normoxia, H = hypoxia.

As shown in Figure 2a,c, treatment with C22 reduced SMO expression under normoxia and more evidently under hypoxia in both cell lines. We detected an effect of GlaB on SMO but it was not significant: a SMO reduction could be ascribed to the fact that in some cellular models SMO could be a GLI1 target gene [21]. Then, we checked GLI1 expression after chemical treatment: GLI1 expression (b–d) was reduced in normoxia and hypoxia by the two chemical compounds and, notably, C22’s reduction effect was more evident and always significant in both cell lines, in particular under hypoxia. As a result, we demonstrated that chemical compounds GlaB and C22 were able to impair GLI1 and SMO expression.

### 2.3. CAXII Protein Level Decreases after Chemical Compound Treatment

**Figure 3:**
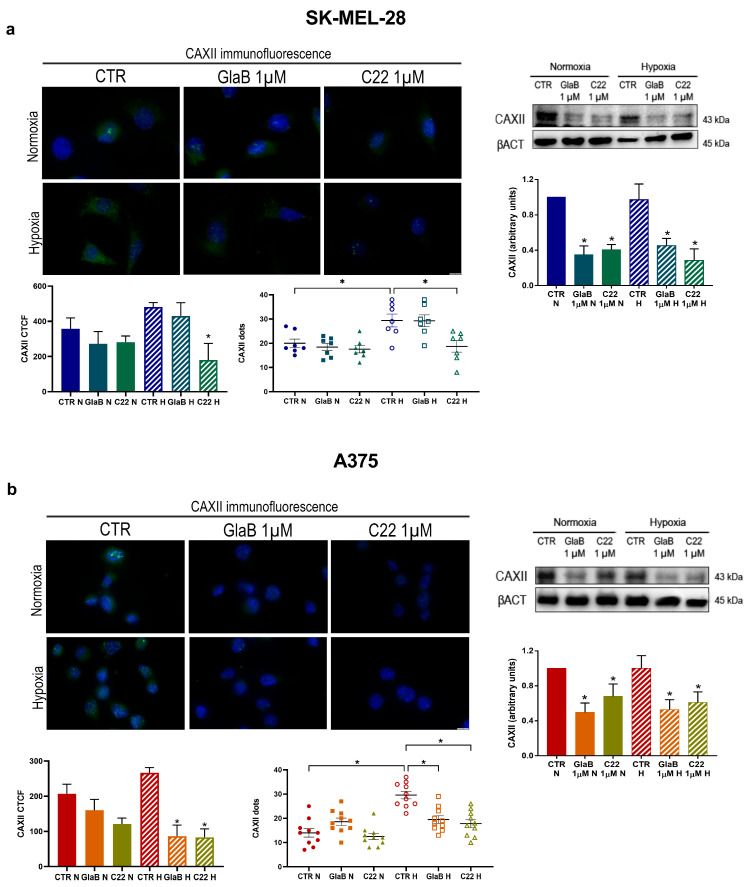
CAXII expression after chemical treatment. CAXII immunofluorescence (60× magnification, scale bar 10 μm) and Western blot after treatment with GlaB or C22 in SK-MEL-28 (**a**) and A375 (**b**) under normoxia and hypoxia for 24 h. CAXII dots were counted in at least 10 cells per condition. β-actin was used as loading control for Western blot. Blots are representative of three independent experiments. Means ± SEM are presented. (*n* = 3; * *p* ≤ 0.05 indicates statistically significant difference). CTR = control, N = normoxia, H = hypoxia.

Furthermore, to see if CAXII expression could be affected by GlaB and C22 treatment we evaluated by Western blot analysis its expression in SK-MEL-28 and A375 treated with these compounds. As shown in Figure 3, inhibition of GLI1 and SMO resulted in a significant downregulation of CAXII in SK-MEL-28 (a) and A375 (b), both under normoxia and hypoxia. To further confirm this effect driven by the two Hh inhibitors, GlaB and C22, we performed an immunofluorescence analysis. As shown here, CAXII protein levels are reduced in both cell lines under normoxia and hypoxia when chemical treatment was performed. Of interest, we detected a spotty appearance of CAXII in both cell lines, due to possible membrane accumulation. We managed to count CAXII dots demonstrating a significant increase in hypoxic conditions in both cell lines and a significant reduction in GlaB- and C22-treated cells under hypoxia: in the A375 cell line, both compounds, GlaB and C22, determined a significant reduction in dots. In conclusion, CAXII expression is impaired by GlaB and C22 under normoxia and hypoxia in both cell lines.

### 2.4. Melanoma Cell Migration Is Impaired by SMO and GLI1 Chemical Inhibitors

**Figure 4:**
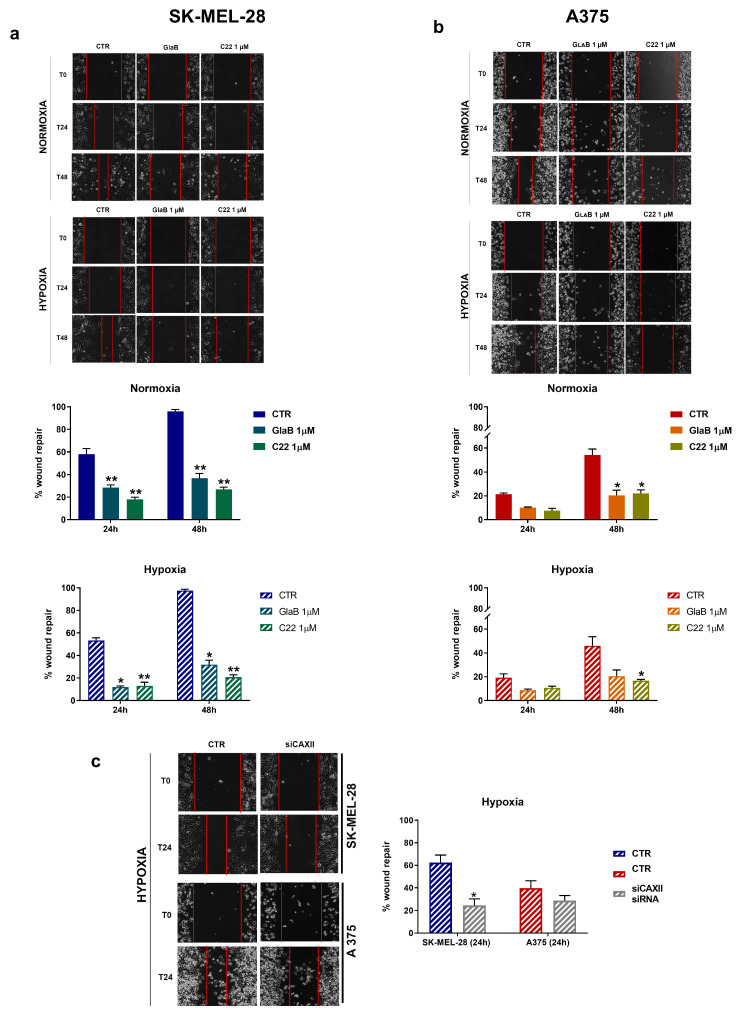
SK-MEL-28 and A375 cell migration was impaired by GlaB and C22. Cell migration measured by wound-healing assays under normoxic or hypoxic conditions in SK-MEL-28 (**a**) and A375 (**b**) treated with GlaB and C22 for 24 h and 48 h (and AraC (2.5 μg/mL)). Pictures are representative of three independent experiments. Means ± SEM are presented. (*n* = 3; * *p* ≤ 0.05 and ** *p* ≤ 0.01 indicate statistically significant differences). (**c**) SK-MEL-28 and A375 cell migration was impaired by CAXII transient knockdown. Cell migration measured by wound-healing assays under hypoxia. Pictures are representative of three independent experiments. Means ± SEM are presented. (*n* = 3; * *p* ≤ 0.05).

Knowing that CAXII expression is related to melanoma cell migration, and having demonstrated that GlaB and C22 chemical compounds impair CAXII, we decided to perform a migration assay to test migration abilities of both cell lines after treatments with the two small molecules (AraC 2.5 μg/mL was added to inhibit proliferation).A wound-healing assay was carried out for 24 and 48 h and, once stabilised, images were acquired. As shown in Figure 4a, SK-MEL-28 migration was significantly impaired by the two compounds both under normoxia and hypoxia. In Figure 4b, we show a reduced migration ability of A375 cells in the presence of the two chemical compounds in normoxia and hypoxia. In the latter experimental condition, the reduced ability of repairing the wound is statistically significant for C22. Of interest, even though the A375 cell line has a reduced migration ability compared to SK-MEL-28, the two small molecules were effective in reducing this aspect mostly in the hypoxic condition.

In addition, we show (panel c) that CAXII siRNA under hypoxia was able, similarly to GlaB and C22, to reduce migration of SK-MEL-28 and A375. We hypothesise that CAXII is involved in the impairment of melanoma cell migration in response to GlaB and C22.

### 2.5. Metalloprotease 9 and 2 Activity Was Reduced after Chemical Treatment

**Figure 5:**
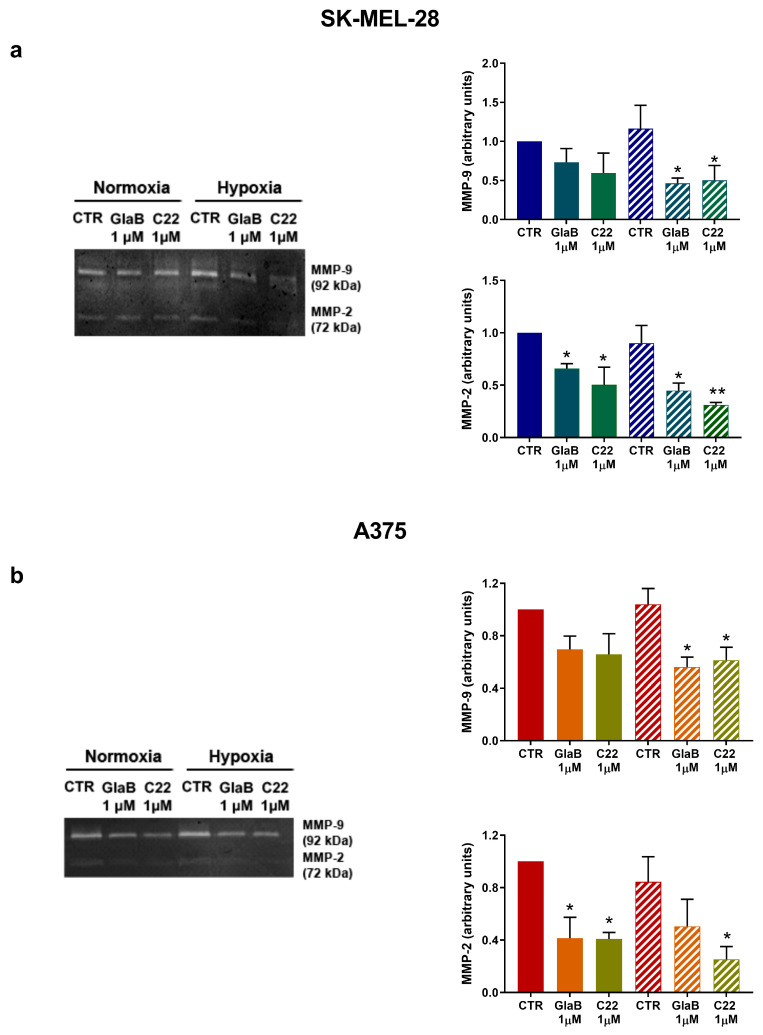
GlaB and C22 reduced MMP-2/MMP-9 activity. Zymogram assays in SK-MEL-28 (**a**) and A375 (**b**) cells in normoxia and hypoxia treated with GlaB and C22 for 24 h. Pictures and blots are representative of three independent experiments. Means ± SEM are presented. (*n* = 3; * *p* ≤ 0.05 and ** *p* ≤ 0.01 indicates statistically significant difference).

Next, as metalloprotease activity is related to melanoma invasion, we observed MMP-9 and MMP-2 in SK-MEL-28 by zymogram assay. In Figure 5a, MMP-9 and MMP-2 activity is higher under hypoxia with respect to normoxia, but we detected a reduced activity when cells were treated with GlaB and C22; notably, under hypoxia both compounds determined a significant reduction of MMP-9 and MMP-2. Following the same experimental workflow, we evaluated MMP-9 and MMP-2 in A375: in Figure 5b, it is shown that GlaB and C22 were able to reduce A375 metalloprotease activity and, of interest, C22 significantly reduced MMP-2 under normoxia and hypoxia.

### 2.6. SK-MEL-28 and A375 Invasion Is Downregulated by GlaB and C22

**Figure 6:**
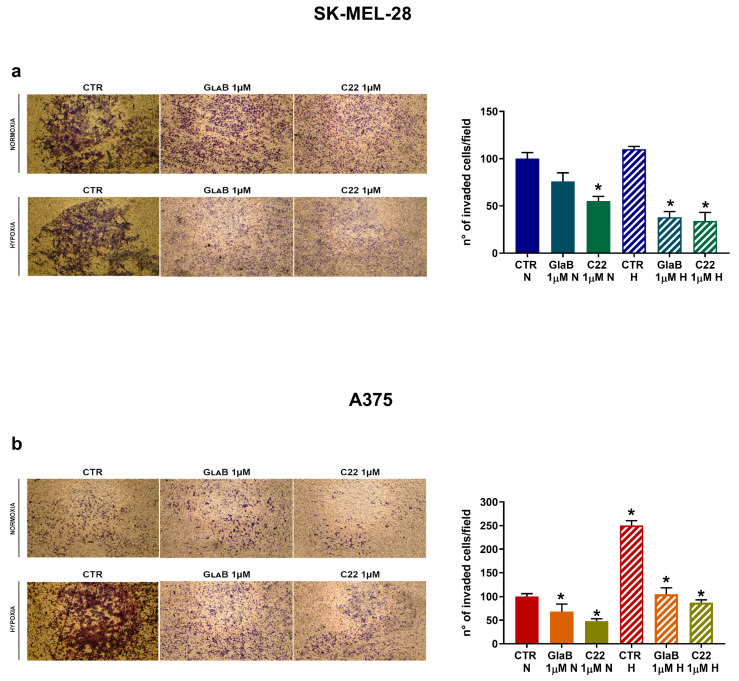
GlaB and C22 treatment reduced melanoma cell invasion. Cell invasion measured by modified Boyden chamber assay in SK-MEL-28 (**a**) and A375 (**b**) cells treated with GlaB and C22 under normoxic and hypoxic conditions for 24 h. Pictures are representative of three independent experiments. Means ± SEM are presented. (*n* = 3; * *p* ≤ 0.05 indicates statistically significant difference).

Finally, we tested the invasion ability of both cell lines by setting up a modified Boyden chamber assay and acquiring images of invaded cells at 24 h in order to observe the effect of GlaB and C22 on melanoma cell invasion. In Figure 6a, we demonstrate that SK-MEL-28 invasion was impaired by GlaB significantly under hypoxia, while C22 significantly reduced the number of invaded cells both in normoxia and hypoxia. In the same way, we evaluated A375 invasion ability, as described in Figure 6b, where under normoxia and hypoxia the two chemical compounds significantly reduced the invasion ability of the more aggressive melanoma cell line (compared to SK-MEL-28). Compared to SK-MEL-28, A375, when exposed to hypoxia, presented a more invasive phenotype, but despite this aspect, chemical treatment with GlaB and C22 was also effective in hypoxia. As a final result, we demonstrated that melanoma cell invasion can be inhibited by chemically targeting the Hh pathway under normoxia and hypoxia.

## 3. Discussion

The Hh pathway is a promising target in cancer treatment. We have previously demonstrated that melanoma migration and invasion can be impaired by siRNA targeting Hh pathway components: siRNA treatment targeting SMO and GLI1 was effective and proved to reduce migration and invasion in two different melanoma cell lines, SK-MEL-28 and A375. However, siRNAs are associated with several druggability challenges, and their development into drugs is still a complex process compared to small molecules [20]. Consequently, in our experimental model we treated these cell lines with two different chemical compounds able to modulate the Hh pathway with different mechanisms of action in order to see if we could obtain a similar outcome to siRNA [16] as a first step for the development of new therapeutical strategies in melanoma treatment.

Firstly, we observed HIF-1α and SMO kinetics under exposure to normoxia and hypoxia, detecting an increase in the two proteins mostly at 24 h and 48 h. We confirmed our hypoxic experimental results by observing an increased HIF-1α level when SK-MEL-28 and A375 were exposed to 2% O_2_; we notice the colocalisation between the HIF-1α signal and cell nucleus in the hypoxic condition, which indicates the presence of HIF-1 as an active transcriptional factor. As a matter of fact, the heterodimer HIF-1α/HIF-1β binds to a DNA sequence called the hypoxia responsive element (HRE), which is necessary for the transcription of its target genes [22,23].

Interestingly, hypoxia exposure was able to significantly increase CAXII expression, showing a relation between CAXII and HIF-1α. As a matter of fact, CAIX, which catalyses the reversible hydration of carbon dioxide (as CAXII), contains in its gene promoter an HRE, so it may be considered a biomarker of tumour hypoxia [24]. Even if there are several putative HRE sequences in the upstream region of the CAXII gene, their functionality has not been studied yet; hypoxia induction of CAXII has also been seen in various tumour cells, but the link between CAXII and hypoxia still remains unknown [25]. In addition, we observed a relation between CAXII and the Hh pathway, as CAXII expression was modulated by cyclopamine (SMO antagonist) and SMO agonist (SAG). Indeed, the Hh pathway interacts with many others signalling pathways, such as Notch and Wnt; this crosstalk causes a synergistic effect observed in tumorigenesis [26].

In this work, to deeply highlight the interplay between CAXII, the Hh pathway and hypoxia, we evaluated the activity of the small molecules GlaB and C22, which act as GLI1 and GLI1/SMO multitarget inhibitors, respectively. CAXII expression was impaired by the chemical compounds in both our experimental conditions and, to see if there was a functional effect on both melanoma cell line migration and invasion abilities, we performed a wound-healing assay and an invasion assay. We observed a reduction in migration and invasion of both melanoma cell lines associated with reduced activity of MMP-9 and MMP-2 when treatment with GlaB and C22 was performed. In previous work [27,28], it has been demonstrated that CAXII expression is related to the Hh pathway, as inhibition of SMO determined its downregulation in breast cancer. In addition, in a previous study phosphorylation of FAK, a protein involved in epithelial-to-mesenchymal transition, was reduced in the presence of carbonic anhydrase inhibitors [17]. In a similar way, these data, taken together, highlight the possible interplay between the Hh pathway and CAXII in melanoma cell lines.

In addition, the immunofluorescence assay of CAXII underlined its spotty presence in both cell lines; dots may be associated with determined plasma membrane accumulation sites or vesicles, indicating a more active intracellular transport of CAXII. Indeed, we were able to distinguish and count CAXII dots, revealing their decrease or increase (depending on treatment conditions) which was parallel to the total CAXII fluorescence signal trend. Others have demonstrated that the cell surface level of CAIX is regulated by the AMAP1-PRKD2 pathway, indicating the presence of a precise recycling activity [29] but further studies will be necessary to understand this aspect regarding CAXII and its recycling. Inhibition of the Hh pathway seems to be a promising target in melanoma; it is reported that an elevated SMO expression is associated with a shorter survival of melanoma patients, while higher GLI3 (Hh pathway repressor) expression is related to better survival [30]. In this context, CAXII protein level can represent a crucial role: when the Hh signal is impaired with different molecules (cyclopamine, GlaB, C22) or, as we previously demonstrated, with siRNA, CAXII expression is reduced. In addition, SK-MEL-28 shows an increased migration ability compared to A375, while A375 presents a more invasive phenotype (enhanced by hypoxia). However, despite this aspect, chemical treatment with GlaB and C22 was even effective in hypoxia in both cell lines.

**Figure 7:**
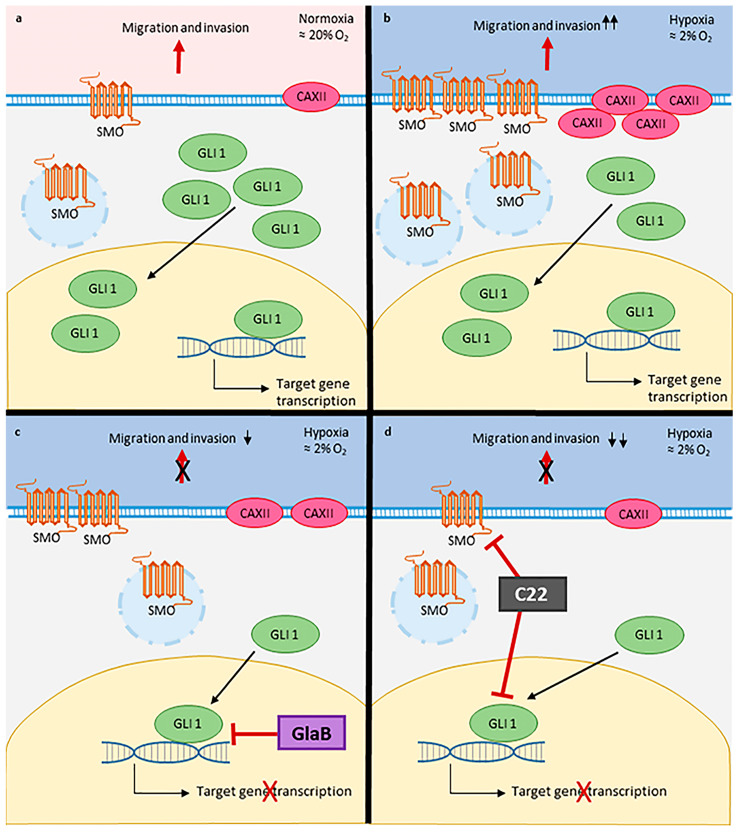
Schematic representation of GlaB and C22 mechanism of action. (**a**) GL1, SMO and CAXII in melanoma cells under normoxia and (**b**) hypoxia. (**c**) Mechanism of action of GlaB and (**d**) of C22 in melanoma cells exposed to hypoxia.

Overall, our findings support the pharmacological relevance of Hh inhibitors acting as GLI1 or SMO/GLI1 multitarget inhibitors, also corroborating the validity of the isoflavone scaffold in the design of effective Hh pathway inhibitors with anticancer potential. It is worth noting that these molecules are devoid of unspecific cytotoxicity, as highlighted by multiple in vitro and in vivo studies, and off-target effects are limited or unpredictable. Indeed, a previous study on a panel of kinase enzymes potentially involved in the regulation of GLI1 functions has shown no effect with the parent compound GlaB at 10 μM concentration, which further reinforces the privileges of exploiting the isoflavone scaffold in the design of Hh inhibitors [18]. Development of new small molecules could be crucial, as evidence of drug resistance is arising in some types of melanoma [31,32] as well as in other Hh-related tumours upon treatment with SMO antagonists. Among all SMO mutations characterised so far [33], the early-induced D473H drug-resistant mutation of SMO in clinical studies by treatment with the SMO antagonist vismodegib represents a case study [34] suggesting that targeting the Hh pathway can be a promising strategy although downstream targets such as GLI1 or multitarget approaches might be effective to circumvent or escape drug resistance. As a matter of fact, development of effective small molecules can be improved and constitute a starting point to elaborate new therapeutical strategies. Actually, a clinical trial or a chemical preparation based on small molecules is less complicated: it is easier to optimise the pharmacokinetics of a chemical compound. In vivo experiments demonstrated that GlaB inhibits glioma cell growth and exacerbates the Warburg effect; by inhibiting GLI1 and lactate efflux at the same time, the antitumour effect in vivo was enhanced, providing a new therapeutical strategy for this brain tumour [35]. Then, with chemical compounds we have the possibility to inactivate a precise binding site on a target protein, and in this work we took into account GLI1 and SMO, so inhibitors of the Hh pathway can also be explored in future research. As shown and simplified in Figure 7, we conclude that CAXII is involved in the effect of GlaB and C22 on melanoma cell migration. Further studies are necessary to better understand the link between CAXII and the Hh pathway. Our data acquire a certain importance in this context because GlaB and especially C22 can be profitable leads in the design of further generations of pharmacological agents targeting the Hh pathway in the therapy of melanoma.

## 4. Materials and Methods

### 4.1. Cell Cultures

SK-MEL-28 and A375 cell lines were supplied by Dr. Francesca Chiarini (University of Bologna, Bologna, Italy) and Prof. Luisa Bracci (University of Siena, Siena, Italy), respectively. SK-MEL-28 was grown with RPMI and A375 with DMEM, both supplemented with antibiotics, L-glutamin and 10% fetal bovine serum (FBS) (Euroclone, Devon, UK). The two cell lines were maintained in a humidified atmosphere at 37 °C, 5% CO_2_, 20% O_2_, or at 37 °C, 5% CO_2_, 2% O_2_ to mimic a hypoxic microenvironment by using the INVIVO_2_ 400 workstation (Ruskinn, Pencoed, UK).

For transient transfection experiments, CTR siRNA sequences were supplied by Sigma-Aldrich (Saint Louis, MO, USA), whereas CAXII siRNA was bought from Ambion (Thermo Fisher Scientific, Cleveland, OH, USA). Cells were transfected with 100 nM siRNA by using a NeonTM transfection system (Invitrogen, Paisley, UK).

### 4.2. Chemical Compounds

Cyclopamine (AlphaAesar, Haverhill, MA, USA) was resuspended in DMSO and used at a final concentration of 20 µM. SAG dihydrochloride (Sigma Aldrich, Saint Louis, MO, USA) was used at a final concentration of 250 nM and was diluted in distilled water (ddH_2_O). Glabrescione B (GlaB) and C22 were prepared according to the synthetic procedure reported previously [19,36]. The structure was unambiguously confirmed through nuclear magnetic resonance (NMR) spectroscopy and by electrospray ionisation–high-resolution mass spectrometry (ESI-HRMS).

### 4.3. Western Bot

SK-MEL-28 and A375 were seeded in Petri dishes, treatments with the compound was of interest were performed and experiments were conducted under normoxia and hypoxia. Cells were lysed with Laemmli buffer with a mixture of proteinase inhibitors (Sigma Aldrich, Saint Louis, MO, USA) in order to carry out a Western blot analysis. Samples were sonicated and the protein level was quantified with a Micro BCA Protein Assay REagent Kit (Thermo Fisher Scientific, Cleveland, OH, USA). Then, 30 μg/lane of proteins was loaded in 10% acrylamide gels, blotted on 0.2 μm nitrocellulose membranes (BIO-RAD, Hercules, CA, USA) and saturation of aspecific binding sites was performed with 5% milk for 1 h. SMO, CAXII (Santa Cruz, Dallas, TX, USA), GLI1 and β-actin (Cell Signaling, Denver, CO, USA) protein expression was detected by incubating membranes with the appropriate primary antibody; antimouse IgG HRP and antirabbit IgG HRP (Cell Signaling Technologies, Danvers, MA, USA) were used as secondary antibodies; images of the chemiluminescent bands were taken with a ChemiDoc™ MP System and analysed by Image Lab software 5.1 (BIO-RAD, Hercules, CA, USA).

### 4.4. Wound-Healing Assay

Both cell lines were seeded in a culture-insert 2 well in a 35 mm µ-Dish (ibidi culture-insert 2 well, ibidi GmbH, Martinsried, Germany). The day after, the culture-insert was removed and non-adherent cells were washed with PBS. New culture medium with 10% FBS, AraC (2.5 μg/mL) and chemical compound was added. Cells were incubated either under normoxia (37 °C, 5% CO_2_, 20% O_2_) or hypoxia (37 °C, 5% CO_2_, 2% O_2_) for 24 h and 48 h. At the established times of 0 h, 24 h, 48 h, images of the wound gap were acquired with a phase-contrast microscope (10×) (Olympus IX81, Tokyo, Japan) and analysed with Cell F© software 11.21 (Olympus, Tokyo, Japan). Migration was calculated as (1 − $\frac{Ax}{A0}$) and data expressed in % of wound repair (*Ax* and *A*0 represent the empty area at the acquisition times).

### 4.5. Zymography

Cells were incubated with GlaB and C22 chemical compounds for 24 h under normoxia and hypoxia and then media were collected and centrifuged at 300× *g* for 5 min. Total protein amount was analysed with a Micro BCA Protein Assay Reagent kit (Thermo Fisher Scientific, Cleveland, OH, USA) and 10 μg of diluted medium sample was resolved by 1% gelatin from porcine skin–10% acrylamide gels and incubated overnight at 37 °C with agitation in developing buffer. The next day, gels were stained with Coomassie R-250 (Sigma Aldrich, Saint Louis, MO, USA) for the detection of enzymatic activity through colorimetric staining, and images were acquired with a ChemiDoc™ MP System and quantified with ImageJ software 1.53K (http://imagej.nih.gov/ij/docs/index.html) (accessed on 5 February 2024).

### 4.6. Modified Boyden Chamber

Boyden 48-well microchemotaxis chambers (Neuro Probe, Gaithersburg, MD, UK) with 8µm pore size polycarbonate polyvinylpyrrolidone-free nucleopore filters, precoated with 100 µL of 0.2 mg/mL Matrigel (Corning, Life Science, Corning, Tewksbury, MA, USA), were used to test melanoma invasion ability. Cells, with or without compound treatments, were seeded in the upper chamber in 50 µL RPMI or DMEM with 0.1% BSA. NIH3T3 supernatant was employed as chemoattractant in the lower chamber. After 24 h, cells were fixed and stained with Diff Quick (Merz-Dade, Düdingen, Switzerland) and images were acquired at 5× magnification with an Olympus IX81 microscope (Tokyo, Japan). Data were expressed as % of invaded cells.

### 4.7. Immunofluorescence

First, 25,000 cells for each condition were seeded into 24-well plates with cover slides 10 mm in diameter and exposed to normoxia or hypoxia for 24 h. Cells were washed with PBS and fixed and permeabilised with methanol at −20 °C. Primary antibodies HIF-1α (BD Biosciences, San Jose, CA, USA), CAXII, SMO (Santa Cruz, Dallas, TX, USA) and GLI1 (Cell Signaling, Denver, CO, USA) were diluted 1:300 in PBS 2% BSA and incubated overnight at 4 °C in a humidified chamber. The next day, after 2 washing steps with PBS 0.2% BSA, secondary antibody antimouse 488 conjugated (Thermo Fisher Scientific, Cleveland, OH, USA) or antirabbit 550 conjugated (Thermo Fisher Scientific, Cleveland, OH, USA, 1:300) was added in each well and incubated at RT for 1 h. Nuclei were stained with Hoechst 33342 (Fluka, Sigma Aldrich, Saint Louis, MO, USA) 1 mg/mL (1:1000) for 4 min at RT; wells were washed with PBS 0.2% BSA, and cover slides were mounted onto glass microscope slides. Images with 20× or 60× magnification were acquired with an Olympus IX81 microscope (Tokyo, Japan) and analysed with ImageJ software 1.53K (http://imagej.nih.gov/ij/docs/index.html) (accessed on 5 February 2024); fluorescence intensity was expressed by corrected total cell fluorescence (CTCF) = integrated density—(area of selected cell X mean fluorescence of background readings); colocalisation analysis was performed with JACoP plugin, calculating Mander’s coefficient [37].

### 4.8. Statistical Analyses

Data are shown as means ± SEM of at least three independent experiments. Statistical analyses were performed using an ANOVA test with GraphPad Prism 7 software (San Diego, CA, USA). Values of *p* ≤ 0.05 and *p* ≤ 0.01 were conventionally considered statistically significant.

## 5. Conclusions

In this work, we tested chemical compounds, GlaB and C22, in two melanoma cell lines and we underlined the interplay between CAXII, hypoxia and the Hh pathway. Indeed, our data showed that chemical targeting of SMO and GLI1 can impair CAXII expression and melanoma migration and invasion, highlighting, in this experimental model, the possibility to develop small molecules that are more effective in reducing melanoma aggressiveness.

## Data Availability

The data generated during the current study are available from the corresponding author on reasonable request.

## References

[1] Rebecca V.W., Sondak V.K., Smalley K.S. (2012). A brief history of melanoma: From mummies to mutations. Melanoma Res..

[2] Teixido C., Castillo P., Martinez-Vila C., Arance A., Alos L. (2021). Molecular Markers and Targets in Melanoma. Cells.

[3] Nazarian R., Shi H., Wang Q., Kong X., Koya R.C., Lee H., Chen Z., Lee M.K., Attar N., Sazegar H. (2010). Melanomas acquire resistance to B-RAF(V600E) inhibition by RTK or N-RAS upregulation. Nature.

[4] Homet B., Ribas A. (2014). New drug targets in metastatic melanoma. J. Pathol..

[5] Davis L.E., Shalin S.C., Tackett A.J. (2019). Current state of melanoma diagnosis and treatment. Cancer Biol. Ther..

[6] Suchors C., Kim J. (2022). Canonical Hedgehog Pathway and Noncanonical GLI Transcription Factor Activation in Cancer. Cells.

[7] Pietrobono S., Gaudio E., Gagliardi S., Zitani M., Carrassa L., Migliorini F., Petricci E., Manetti F., Makukhin N., Bond A.G. (2021). Targeting non-canonical activation of GLI1 by the SOX2-BRD4 transcriptional complex improves the efficacy of HEDGEHOG pathway inhibition in melanoma. Oncogene.

[8] Teperino R., Aberger F., Esterbauer H., Riobo N., Pospisilik J.A. (2014). Canonical and non-canonical Hedgehog signalling and the control of metabolism. Semin. Cell Dev. Biol..

[9] Robbins D.J., Fei D.L., Riobo N.A. (2012). The Hedgehog signal transduction network. Sci. Signal..

[10] Carballo G.B., Honorato J.R., de Lopes G.P.F., Spohr T. (2018). A highlight on Sonic hedgehog pathway. Cell Commun. Signal..

[11] Gurzu S., Beleaua M.A., Jung I. (2018). The role of tumor microenvironment in development and progression of malignant melanomas—A systematic review. Rom. J. Morphol. Embryol..

[12] Muz B., de la Puente P., Azab F., Azab A.K. (2015). The role of hypoxia in cancer progression, angiogenesis, metastasis, and resistance to therapy. Hypoxia.

[13] Semenza G.L. (2000). Hypoxia, clonal selection, and the role of HIF-1 in tumor progression. Crit. Rev. Biochem. Mol. Biol..

[14] Supuran C.T. (2018). Carbonic anhydrases and metabolism. Metabolites.

[15] Akocak S., Supuran C.T. (2019). Activation of alpha-, beta-, gamma- delta-, zeta- and eta- class of carbonic anhydrases with amines and amino acids: A review. J. Enzym. Inhib. Med. Chem..

[16] Giuntini G., Coppola F., Falsini A., Filippi I., Monaci S., Naldini A., Carraro F. (2022). Role of the Hedgehog Pathway and CAXII in Controlling Melanoma Cell Migration and Invasion in Hypoxia. Cancers.

[17] Giuntini G., Monaci S., Cau Y., Mori M., Naldini A., Carraro F. (2020). Inhibition of Melanoma Cell Migration and Invasion Targeting the Hypoxic Tumor Associated CAXII. Cancers.

[18] Infante P., Mori M., Alfonsi R., Ghirga F., Aiello F., Toscano S., Ingallina C., Siler M., Cucchi D., Po A. (2015). Gli1/DNA interaction is a druggable target for Hedgehog-dependent tumors. EMBO J..

[19] Lospinoso Severini L., Quaglio D., Basili I., Ghirga F., Bufalieri F., Caimano M., Balducci S., Moretti M., Romeo I., Loricchio E. (2019). A smo/gli multitarget hedgehog pathway inhibitor impairs tumor growth. Cancers.

[20] Guo S., Zhang M., Huang Y. (2023). Three ‘E’ challenges for siRNA drug development. Trends Mol. Med..

[21] Doheny D., Manore S.G., Wong G.L., Lo H.W. (2020). Hedgehog Signaling and Truncated GLI1 in Cancer. Cells.

[22] Wang G.L., Jiang B.H., Rue E.A., Semenza G.L. (1995). Hypoxia-inducible factor 1 is a basic-helix-loop-helix-PAS heterodimer regulated by cellular O2 tension. Proc. Natl. Acad. Sci. USA.

[23] Masoud G.N., Li W. (2015). HIF-1alpha pathway: Role, regulation and intervention for cancer therapy. Acta Pharm. Sin. B.

[24] Tafreshi N.K., Lloyd M.C., Proemsey J.B., Bui M.M., Kim J., Gillies R.J., Morse D.L. (2016). Evaluation of CAIX and CAXII Expression in Breast Cancer at Varied O_2_ Levels: CAIX is the Superior Surrogate Imaging Biomarker of Tumor Hypoxia. Mol. Imaging Biol..

[25] Wykoff C.C., Beasley N.J., Watson P.H., Turner K.J., Pastorek J., Sibtain A., Wilson G.D., Turley H., Talks K.L., Maxwell P.H. (2000). Hypoxia-inducible expression of tumor-associated carbonic anhydrases. Cancer Res..

[26] Xia R., Xu M., Yang J., Ma X. (2022). The role of Hedgehog and Notch signaling pathway in cancer. Mol. Biomed..

[27] Guerrini G., Criscuoli M., Filippi I., Naldini A., Carraro F. (2018). Inhibition of smoothened in breast cancer cells reduces CAXII expression and cell migration. J. Cell Physiol..

[28] Guerrini G., Durivault J., Filippi I., Criscuoli M., Monaci S., Pouyssegur J., Naldini A., Carraro F., Parks S.K. (2019). Carbonic anhydrase XII expression is linked to suppression of Sonic hedgehog ligand expression in triple negative breast cancer cells. Biochem. Biophys. Res. Commun..

[29] Horikawa M., Sabe H., Onodera Y. (2022). Dual roles of AMAP1 in the transcriptional regulation and intracellular trafficking of carbonic anhydrase IX. Transl. Oncol..

[30] O’Reilly K.E., De Miera E.V.-S., Segura M.F., Friedman E., Poliseno L., Han S.W., Zhong J., Zavadil J., Pavlick A., Hernando E. (2013). Hedgehog pathway blockade inhibits melanoma cell growth in vitro and in vivo. Pharmaceuticals.

[31] Hossain S.M., Eccles M.R. (2023). Phenotype Switching and the Melanoma Microenvironment; Impact on Immunotherapy and Drug Resistance. Int. J. Mol. Sci..

[32] Winder M., Viros A. (2018). Mechanisms of Drug Resistance in Melanoma. Handb. Exp. Pharmacol..

[33] Atwood S.X., Sarin K.Y., Whitson R.J., Li J.R., Kim G., Rezaee M., Ally M.S., Kim J., Yao C., Chang A.L. (2015). Smoothened variants explain the majority of drug resistance in basal cell carcinoma. Cancer Cell.

[34] Yauch R.L., Dijkgraaf G.J., Alicke B., Januario T., Ahn C.P., Holcomb T., Pujara K., Stinson J., Callahan C.A., Tang T. (2009). Smoothened mutation confers resistance to a Hedgehog pathway inhibitor in medulloblastoma. Science.

[35] D’Alessandro G., Quaglio D., Monaco L., Lauro C., Ghirga F., Ingallina C., De Martino M., Fucile S., Porzia A., Di Castro M.A. (2019). (1)H-NMR metabolomics reveals the Glabrescione B exacerbation of glycolytic metabolism beside the cell growth inhibitory effect in glioma. Cell Commun. Signal..

[36] Berardozzi S., Bernardi F., Infante P., Ingallina C., Toscano S., De Paolis E., Alfonsi R., Caimano M., Botta B., Mori M. (2018). Synergistic inhibition of the Hedgehog pathway by newly designed Smo and Gli antagonists bearing the isoflavone scaffold. Eur. J. Med. Chem..

[37] Manders E.M., Stap J., Brakenhoff G.J., van Driel R., Aten J.A. (1992). Dynamics of three-dimensional replication patterns during the S-phase, analysed by double labelling of DNA and confocal microscopy. J. Cell Sci..

